# Damage Fracture Characterization of Asphalt Mixtures Considering Freeze–Thaw Cycling and Aging Effects Based on Acoustic Emission Monitoring

**DOI:** 10.3390/ma14205930

**Published:** 2021-10-09

**Authors:** Liuxu Fu, Huanyun Zhou, Jing Yuan, Weiliang An, Xianhua Chen

**Affiliations:** 1 School of Transportation, Southeast University, Nanjing 211189, China; fulx@seu.edu.cn (L.F.); zhyjy@seu.edu.cn (H.Z.); awl9817@163.com (W.A.); 2 Broadvision Engineering Consultants, Kunming 650011, China; yuanjing_bec@163.com

**Keywords:** asphalt mixture, fracture behavior, freeze–thaw cycles, aging characteristics, acoustic emission

## Abstract

Freeze–thaw (F–T) cycling and aging effects are the main factors contributing to the deterioration of asphalt mixtures. The acoustic emission (AE) technique enables real-time detection regarding the evolution of internal damage in asphalt mixtures during the loading process. This study set out to investigate the effects of F–T cycling and aging on the damage characteristics of asphalt mixture under splitting loads. Firstly, the Marshall specimens were prepared and then exposed to various numbers of F–T cycles (one, three, five, and seven) and different durations of aging (short-term aging and long-term aging for 24, 72, 120 and 168 h), after which the specimens were loaded by means of indirect tensile (IDT) testing, and corresponding parameters were synchronously collected by the AE acquisition system during the fracture process. Finally, the energy, cumulative energy and peak frequency were selected to investigate the damage mechanisms of asphalt mixtures. The findings demonstrate that the AE parameters provided effective identification of the deterioration for all specimens in real-time, and that the F–T cycling and aging effects altered the damage characteristics of asphalt mixtures, causing early damage, exacerbating the formation of micro-cracks in the early stage, accelerating the expansion of macro-cracks and advancing the debonding between the asphalt and aggregates. The findings of this study provide further insight into the mechanism of F–T cycling and aging effects on the deterioration of asphalt mixture.

## 1. Introduction

Throughout the service course of asphalt pavement, owing to the challenging service environment and the increasing traffic loads, a growing number of pavement diseases occur, which significantly shorten the operational lifespan of the pavement [1,2]. The environmental factors of freeze–thaw (F–T) cycling and aging effects significantly weaken the performance of asphalt pavement [3,4]. Clarifying the deterioration mechanism of asphalt mixture subjected to freeze–thaw cycling and aging effects is essential to prolonging the operational lifespan of asphalt pavement pertinently.

In the seasonally frozen zone, asphalt pavement is subjected to frequent freeze–thaw (F–T) cycles. Following repeated F–T cycling effects, asphalt pavement is affected by moisture and temperature and accumulates internal damage, gradually demonstrating raveling, stripping, potholes and other diseases, and the performance of the asphalt pavement will also be deteriorated to varying degrees [5,6,7]. Many investigations have been conducted on the F–T damage characteristics by analyzing the properties of asphalt mixtures before and after the F–T cycling effect. Xu et al. [8] employed digital image scanning technology to assess the evolution of the internal structure of the asphalt mixture before and after the F–T cycles. Wang et al. [9] demonstrated that the basalt fibers could enhance the resistance of asphalt mixtures against F–T failure by adding 0.4% basalt fibers to asphalt mixture. Fakhri et al. [10] discussed the effect of the counts of F–T cycles (1, 3, 5, 7, and 9) on the cracking characteristics of asphalt mixtures based on fracture mechanics through semi-circular bending (SCB) tests. Karimi et al. [11] proposed an index to evaluate the cracking resistance of asphalt mixtures after F–T cycles in terms of the area enclosed by fracture energy and service time. Gao et al. [12] explored the effect of F–T cycling on the thermal conductivity and water permeability for asphalt mixtures with different gradations.

The aging of asphalt pavement refers to the deterioration of its performance due to irreversible physical and chemical variations caused by exterior environmental agents in terms of moisture, sunlight and oxygen during the service process [13,14]. The aging of an asphalt mixture generally results in an increase in high-temperature rutting resistance, but a significant reduction in moisture stability, low-temperature workability and fatigue resistance, which exacerbates the generation of various diseases on asphalt pavement [15,16]. There has been extensive research into the effect of aging on asphalt mixtures by analyzing the variations in the mechanical parameters before and after aging conditions. Beak et al. [17] explored the influence of aging on the fatigue properties of asphalt mixtures through four different levels of aging tests. Sirin et al. [18] improved the existing long-term aging test method based on the actual climate environment in Qatar through the results of dynamic modulus tests. Lv et al. [19] proposed a viscoelastic model for the fatigue damage of asphalt mixtures that incorporated the aging effect based on the direct tensile fatigue test. Geng et al. [20] addressed the influence of regenerating agents on a rubber-modified asphalt mixture after initial and secondary aging. Amani et al. [21] discussed the influence of different long-term aging levels (3, 5, 7 and 9 days) upon the recovery process of a self-healing asphalt mixture, and concluded that the healing efficiency of the asphalt mixture gradually decreased as the aging time increased. Additionally, some research has been conducted on the aging and temperature effects of different additives on asphalt mixture, such as fly ash [22], basalt fiber and diatomite [23], and copper slag [24].

The above studies focusing on the F–T cycling and aging effects of asphalt mixture were limited to comparing the mechanical parameters before and after the damage, and the failure process of asphalt mixture subjected to F–T cycling and aging cannot be monitored in real-time. The application of acoustic emission (AE) technology can provide further insights into the damage characteristics of asphalt mixture after F–T cycling and aging. AE is a phenomenon of instantaneous elastomeric waves caused by the quick emission of local energy, and is generated when irreversible alterations occur in materials, for example due to plastic deformation or crack formation during the loading process [25,26]. AE technology is widely utilized for the damage evaluation of concrete and rock materials during the loading process due to its real-time dynamic monitoring feature [27,28,29], but has rarely been employed to address the damage fracture mechanisms of asphalt mixtures. Li et al. [30,31] explored the availability of using the AE technique to characterize the cracking mechanism of asphalt mixtures. Arnold et al. [32] investigated the cracking characteristics of a recycled asphalt mixture via the AE technique. Qiu et al. [33,34] proposed a method for locating damage sources of specimens during the failure process based on the propagation features of an AE signal. Jiao et al. [35,36] employed AE parameters to discuss the cracking characteristics as well as the fracture modes of a porous asphalt mixture. Cai et al. [37] estimated the fracture evolution of semi-flexible pavement materials using AE parameters. The aforementioned studies on the utilization of AE technique to monitor the internal damage in asphalt mixtures rarely considered the F–T cycling and aging effects, and the AE technique enables real-time monitoring of damage, which can further clarify the damage characteristics of asphalt mixtures after F–T and aging conditions.

In the present study, Marshall specimens were first fabricated and then exposed to various numbers of F–T cycles (one, three, five and five) and various durations of aging (short-term aging and long-term aging for 24, 72, 120 and 168 h), followed by indirect tensile (IDT) tests, and the AE parameters released during the fracture course of specimens were collected simultaneously. Finally, the energy, cumulative energy and peak frequency were selected to explore the influence of F–T cycling and aging effects on the damage characteristics of the asphalt mixture.

## 2. Materials and Methods

### 2.1. Raw Materials and Specimens

**Figure 1:**
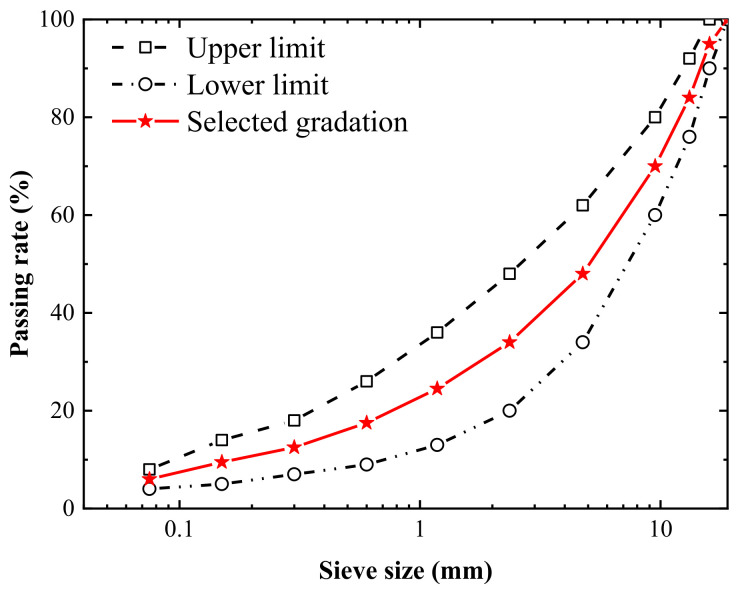
Gradation curve of AC-16.

To improve the high-temperature rutting resistance and low-temperature cracking resistance of conventional asphalt mixture, the asphalt selected in the present study was SBS modified asphalt, produced by Baoli International Investment Co. Ltd., Wuxi, China, whose technical specifications are displayed in Table 1, referring to JTG E42-2005 [38]. The aggregates and mineral powder were all from basalt (Shandong Zhanfei Consturction Material Co. Ltd., Binzhou, China), and the corresponding indicators are listed in Table 2, referring to JTG E20-2011 [39]. The dense-graded AC-16 was selected to fabricate specimens, and the relative gradation curve is illustrated in Figure 1, following JTG F40-2004 [40]. The specimens were prepared according to the Marshall mix design method by compacting 75 stokes on each side, with a height of 63.5 mm and a diameter of 101.6 mm. The optimal asphalt content of AC-16 was 4.8%, which was determined by the variations of the Marshall design parameters.

### 2.2. Testing Procedure

#### 2.2.1. Freeze–Thaw Testing Procedure

The prepared Marshall specimens were placed under vacuum for 15 min, and then the specimens were saturated with water for 30 min. For freezing, the specimens were maintained at −18 °C for 16 h, and for thawing, the specimens were maintained at 60 °C for 8 h, which ended as one freeze–thaw cycle. To investigate the effect of the number of F–T cycles on the damage characteristics of the asphalt mixture, combining existing research on the F–T cycles testing method [10], the specimens were treated with 1, 3, 5 and 7 F–T cycles in accordance with the above testing method for subsequent analysis.

#### 2.2.2. Aging Test Procedure

The aging of the asphalt mixture is categorized as short-term aging and long-term aging. For the short-term aging test, the mixed asphalt mixture was loosely and evenly laid and then maintained inside an oven at 135 °C for 4 h in a ventilated environment, after which it was taken out to fabricate Marshall specimens. For the long-term aging test, to explore the effect of aging time on the damage characteristics of asphalt mixture, in conjunction with existing research on the long-term aging testing method [21], the specimens were firstly compacted after short-term aging and then placed in an oven at 85 °C for 24, 72, 120 and 168 h, respectively, before being taken out for subsequent testing.

#### 2.2.3. Acoustic Emission and Indirect Tensile Tests Procedure

**Figure 2:**
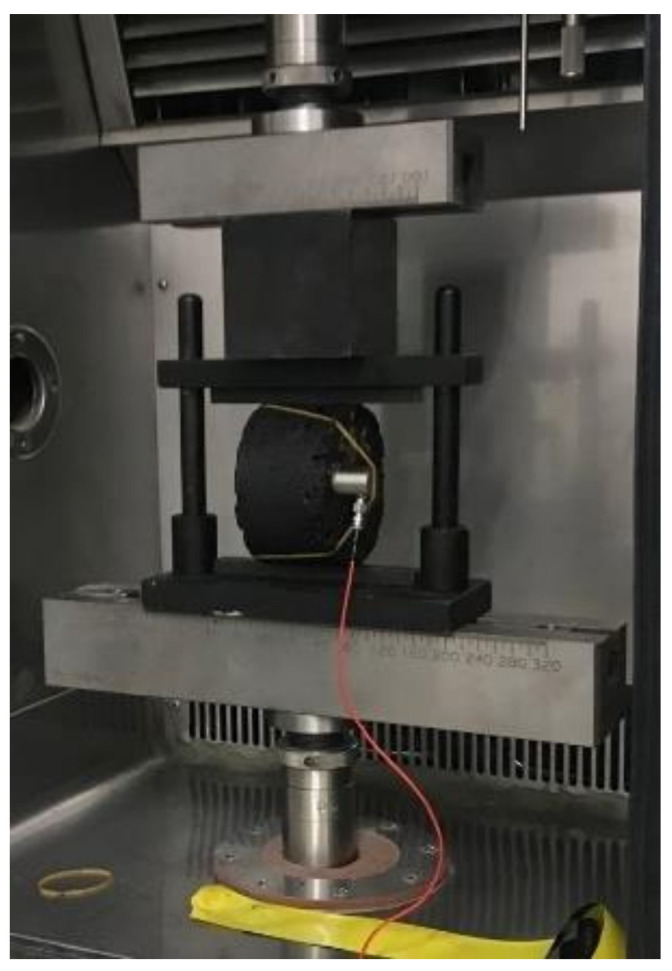
The indirect tensile test of specimen.

**Figure 3:**
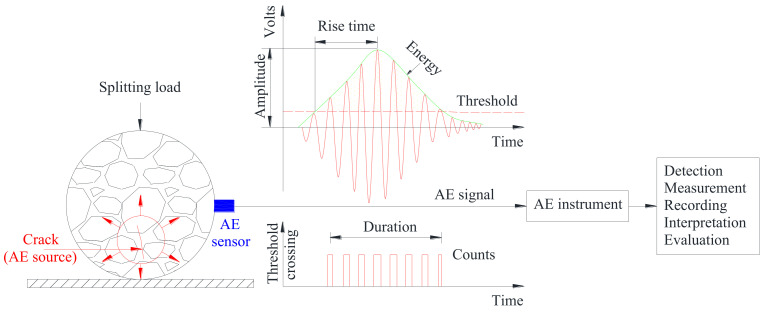
Schematic diagram of indirect tensile test for asphalt mixture monitored by AE.

The prepared specimens were firstly kept at 25 °C for 6 h to guarantee that the asphalt mixture achieved the required temperature internally, then the specimens were placed in the fixture for the IDT test and loaded by a universal testing machine (Sinotest Co. Ltd., Changchun, China) at a rate of 1 mm/min, as shown in Figure 2. Prior to the IDT test, an AE transducer, whose operating frequency ranged from 35 to 400 kHz, was coupled to the lateral center of the cylindrical specimen by means of a lubricant and fixed with an elastic band. Finally, the IDT test was carried out simultaneously with the collection of AE parameters for asphalt mixture during the damage process via the AE acquisition system (Physical Acoustics Corporation, PCI-2,Princeton Jct, NJ, USA). The threshold value was set to 45 dB to filter out the background noise during the acquisition process. To ensure a satisfactory coupling stage between the sensor and the specimen, a lead break test was required prior to the loading. The whole testing procedure is shown in Figure 3. In this study, three parallel tests were performed for each group to obtain their mechanical and acoustic emission parameters. Due to the high consistency of the AE data from the three groups, one of them was selected for subsequent analysis.

### 2.3. AE Parameters

The asphalt mixture generated cracks and released AE signals during the IDT process, which were captured by the AE acquisition system, including energy, rise time, duration, count, amplitude and peak frequency, etc. It has been demonstrated that these parameters varied in a similar manner [35]; therefore, the energy and peak frequency were selected in this study to investigate the fracture characteristics of specimens under different F–T cycles and aging conditions. The energy is determined by the area covered by the count event envelope, reflecting the intensity of the damage [30]. The peak frequency is defined as the frequency associated with the maximum amplitude in the frequency domain waveform [41]. During the loading process of the specimens, the peak frequency usually forms several frequency bands parallel to each other to describe different damage characteristics.

## 3. Results and Discussions

### 3.1. Effect of Freeze–Thaw Cycling on the Damage Mechanisms of the Asphalt Mixture

#### 3.1.1. Failure Loads and Failure Strains

**Figure 4:**
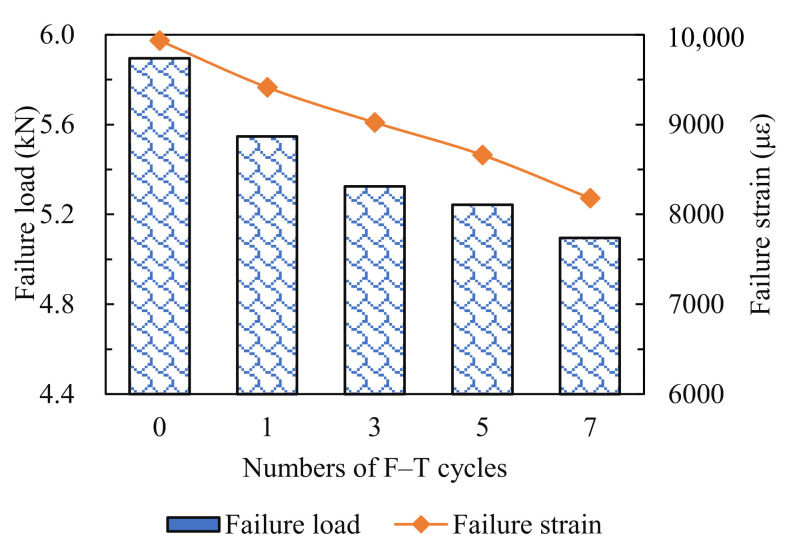
Failure loads and failure strains of specimens.

The failure loads and failure strains under IDT at 25 °C are presented in Figure 4. It can be seen that the failure loads and failure strains of the specimens reduced with the increase in the number of F–T cycles. Specifically, after one, three, five and seven F–T cycles, the failure load was reduced by 5.9%, 9.7%, 11.1% and 13.6%, respectively, and the failure strain of the asphalt mixture was reduced by 5.2%, 9.2%, 12.8% and 17.7%, respectively. This indicated that the F–T cycling effect weakened the mechanical performance of specimens to resist the loading. Nevertheless, this result cannot reveal the real-time fracture mechanisms of specimens during the IDT process, and the AE tests need to be conducted in order to monitor the fracture process of asphalt mixture, which experienced different freeze–thaw cycles.

#### 3.1.2. Energy

**Figure 5:**
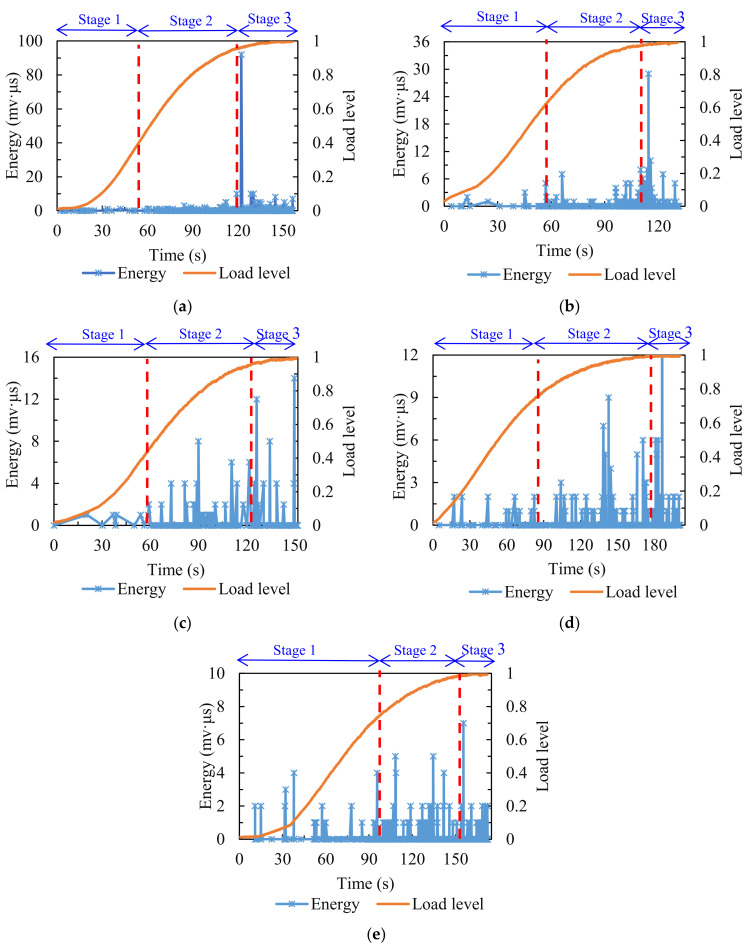
Energy distribution of specimens treated with various numbers of F–T cycles: (**a**) 0; (**b**) 1; (**c**) 3; (**d**) 5; (**e**) 7.

The variations of energy value are presented in Figure 5. The fracture process of the asphalt mixture experiencing different numbers of F–T cycles can be subdivided into three stages. At stage one, for the comparison group (0 F–T cycles), the energy value was close to the axis. After F–T cycling, the energy exhibited a few mutation points at this stage, which became more intensive and frequent with the increase in the number of F–T cycles. This stage was associated with the formation of micro-cracks, and the above results demonstrate that the F–T cycling effect exacerbated the generation of micro-cracks in the asphalt mixture under splitting load, and the greater the number of F–T cycles the sample was subjected to, the more intense the formation of micro-cracks was. At stage two, the energy value of the comparison group was at a low level, near zero. After various numbers of F–T cycles, the surge points in energy value appeared frequently, and this became noticeable when the number of F–T cycles reached three. This stage was concerned with the propagation and expansion of micro-cracks in the asphalt mixture into macro-cracks. The variations in energy value indicated that the F–T cycling effect sped up the process of internal micro-cracks expanding into macro-cracks under splitting load. At stage three, there was a remarkable sudden increase in the energy value of the comparison group. After experiencing a varying number of F–T cycles, the energy value also exhibited a few sudden increases. This stage was related to the accumulation of macro-cracks within the specimen, leading to the final fracture. In terms of the magnitude of the energy released, the higher the energy value, the more intense the internal damage [42]; the comparison group showed the highest energy extreme, much higher than the other groups that experienced F–T cycling, but the release of energy burst points became denser and more frequent after F–T cycling at the first two stages. This indicated that the F–T cycling effect advanced the internal damage of the asphalt mixture.

#### 3.1.3. Cumulative Energy

**Figure 6:**
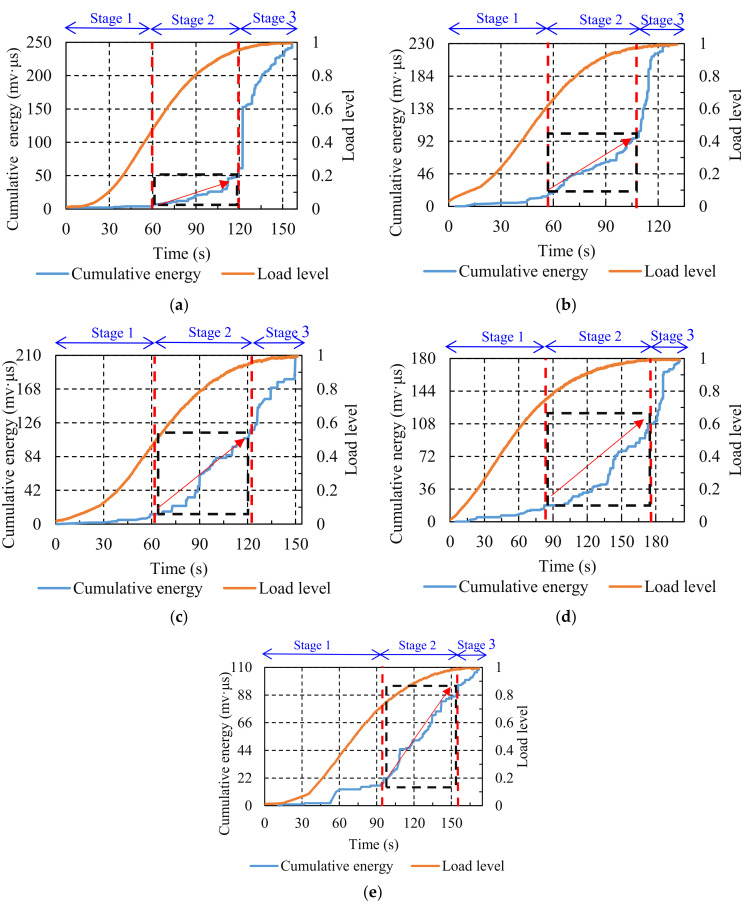
Cumulative energy distribution of specimens treated with various numbers of F–T cycles: (**a**) 0; (**b**) 1; (**c**) 3; (**d**) 5; (**e**) 7. (Red arrow: rising speed of the curves.)

The variations of cumulative energy against loading time are illustrated in Figure 6. The fracture process of the asphalt mixture experiencing different numbers of F–T cycles can be separated into three stages. At stage one, for the comparison group, the cumulative energy curve was close to the horizontal axis. After the F–T cycling, the cumulative energy curve displayed an upward trend, which became more pronounced after the number of F–T cycles reached five. As mentioned earlier, this stage was related to the formation of micro-cracks, and the variations in the cumulative energy curve revealed that the F–T cycling effect promoted the formation of micro-cracks, and this promotion became more significant as the number of F–T cycles increased. At stage two, the cumulative energy curve of the comparison group exhibited a gradual upward trend. The cumulative energy curve also displayed a rising trend after various numbers of F–T cycles, and the curve rose more rapidly as the number of F–T cycles increased. The micro-cracks within the asphalt mixture expanded and developed into macro-cracks at this stage under the splitting load. The variations in the cumulative energy curve indicated that the F–T cycling effect accelerated the evolution of micro-cracks into macro-cracks. At stage three, there was a significant straight upward trend in the cumulative energy curve for the comparison group. After experiencing F–T cycling, a steep upward trend in the cumulative energy curve could also be observed, but the slope of the curve gradually decreased as the counts of F–T cycles increased, dropping to a minimum when the number of F–T cycles reached seven. This stage was considered to be the interconnection and rapid accumulation of macro-cracks within the asphalt mixture, leading to the final fracture under splitting load. The above results demonstrate that the F–T cycling effect advanced the damage to the specimens and weakened the significance of the final brittle fracture.

#### 3.1.4. Peak Frequency

**Figure 7:**
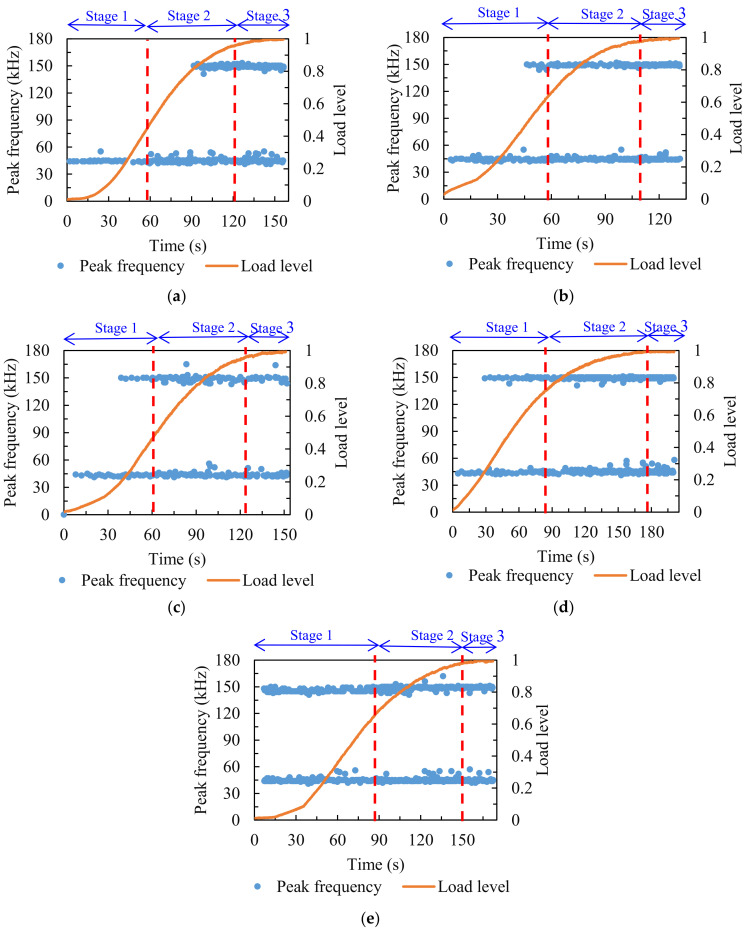
Peak frequency distribution of specimens treated with various F–T cycles: (**a**) 0; (**b**) 1; (**c**) 3; (**d**) 5; (**e**) 7.

The peak frequency distribution of the asphalt mixture is illustrated in Figure 7. For comparative analysis, the stages of the damage process were divided in accordance with the analysis of energy and cumulative energy. It can be seen that there were two distinct peak frequency bands during the fracture process of specimens—the low value of peak frequency (around 45 kHz) and the high value of peak frequency (around 150 kHz). It has previously been illustrated that the peak frequency of the AE signal from matrix cracking was lower than that from debonding [43]. Therefore, in this study, it can be assumed that the peak frequency near 45 kHz represented the cracking of the matrix in the asphalt mixture, and the peak frequency near 150 kHz represented cracking in the asphalt mixture caused by the debonding of the matrix from the aggregates.

For the comparison group, the peak frequency around 45 kHz was accompanied by the entire loading process, and the peak frequency around 150 kHz began to appear from the middle of stage two. After F–T cycling, the peak frequency around 45 kHz still occurred throughout the loading process, but as the counts of F–T cycles increased, the peak frequency around 150 kHz appeared earlier, and it emerged at the beginning of the loading process when the number of F–T cycles reached seven. The variations of peak frequency indicated that the micro-cracks from matrix cracking accompanied the entire loading process, and that the F–T cycling effect weakened the adhesion between the matrix and aggregates, resulting in the micro-cracks within the asphalt mixture due to insufficient adhesion of the matrix debonding from the aggregates at the initial loading process.

The comprehensive energy, cumulative energy and peak frequency analysis results reveal that the F–T cycling effect altered the fracture characteristics of specimens. For the comparison group, the damage characteristics were represented by sudden significant damage at the final loading stage. After the F–T cycling, the asphalt mixture exhibited early damage under splitting load, the formation of micro-cracks was intensified, the expansion and accumulation of macro-cracks were accelerated and the debonding between the matrix and aggregates was advanced.

### 3.2. Effect of Aging on Damage Mechanisms of Asphalt Mixture

#### 3.2.1. Failure Loads and Failure Strains

**Figure 8:**
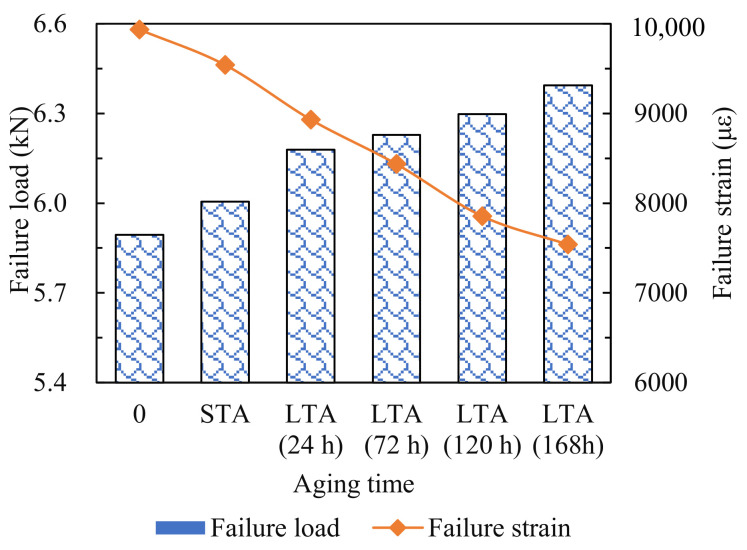
Failure loads and failure strains of specimens.

The failure loads and failure strains of asphalt mixtures treated with various aging conditions are illustrated in Figure 8. As the aging time increased, the failure load increased, while the failure strain decreased. Specifically, compared to the comparison group (0 h), the failure load of specimens increased by 1.8% after short-term aging (STA), and increased by 4.8%, 5.6%, 6.8% and 8.5% after 24, 72, 120 and 168 h of long-term aging (LTA), respectively; the failure strain of specimens decreased by 4.0% after short-term aging, and decreased by 10.1%, 15.1%, 20.9% and 24.1% after 24, 72, 120 and 168 h of long-term aging, respectively. This indicated that aging caused the asphalt to become brittle and weakened the ability of specimens to resist deformation. The above results only reflect the overall mechanical properties of specimens under different aging conditions, and AE tests are required to monitor the failure process of specimens in real-time to capture its damage characteristics.

#### 3.2.2. Energy

**Figure 9:**
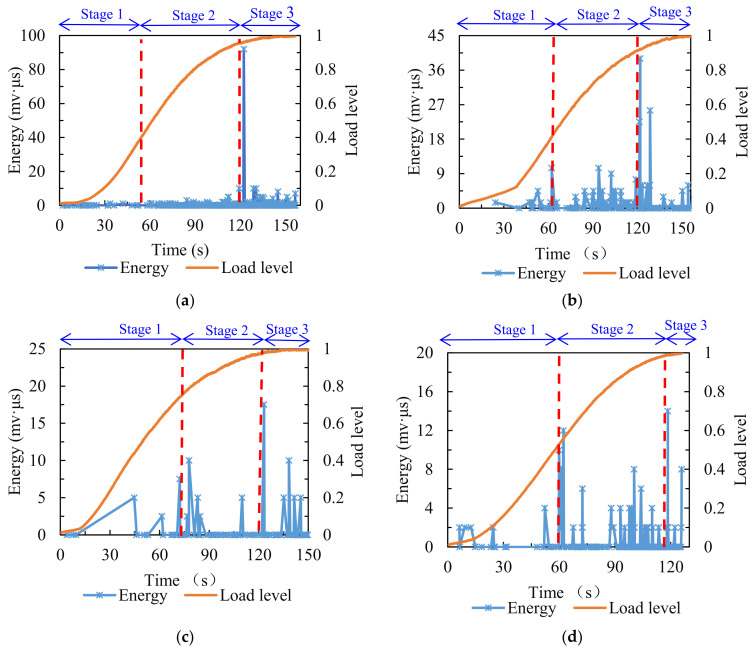

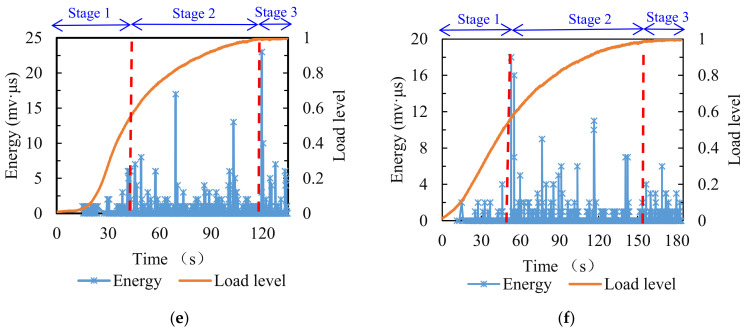
Energy distribution of specimens treated with various aging conditions: (**a**) 0 h; (**b**) STA; (**c**) LTA of 24 h; (**d**) LTA of 72 h; (**e**) LTA of 20 h; (**f**) LTA of 168 h.

The energy distribution of asphalt mixture subjected to various aging times is presented in Figure 9. It can be seen that the failure process of specimens can be segmented into three stages. At stage one, for the comparison group (0 h), the energy value was close to zero. After short-term aging, the energy value appeared more intensively and with a larger value compared to the comparison group. As the long-term aging time increased, the energy was released more densely. This stage was associated with the formation of micro-cracks in asphalt mixture, and the above results indicate that the aging effect aggravated the formation of micro-cracks in specimens at the initial stage of failure process. At stage two, the energy value of the comparison group was still at a low level. After short-term aging, the energy value was at a higher level compared to the comparison group. With the increase in the duration of long-term aging, the release of energy became more intensive and the energy mutation points appeared more frequently. During this stage, the micro-cracks within the asphalt mixture expanded and penetrated into macro-cracks. This demonstrated that the aging effect accelerated the development of macro-cracks within asphalt mixture. At stage three, there was a significant sudden increase in the energy value of the comparison group. After short-term aging, there were two remarkable abrupt increases in energy value. After various long-term aging times, a few noticeable sudden increases in energy value could still be observed, except when the aging time reached 168 h. This stage was related to the final fracture of specimens, and in terms of the extreme value of energy, the largest value was found in the comparison group, followed by the short-term aging group, and further reduced after long-term aging. The above results demonstrate that the aging effect brought forward the release of energy in the final stage, resulting in early damage to the asphalt mixture.

#### 3.2.3. Cumulative Energy

**Figure 10:**
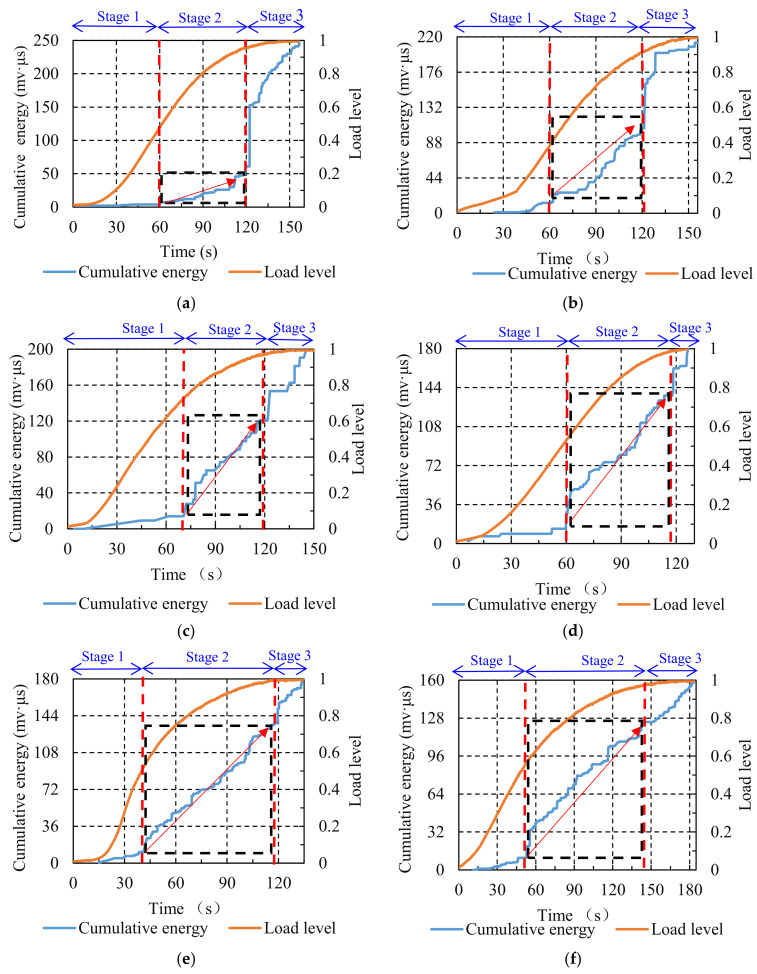
Cumulative energy distribution of specimens treated with various aging conditions: (**a**) 0 h; (**b**) STA; (**c**) LTA of 24 h; (**d**) LTA of 72 h; (**e**) LTA of 20 h; (**f**) LTA of 168 h. (Red arrow: rising speed of the curves.).

The variations of cumulative energy for the asphalt mixture subjected to different aging times are displayed in Figure 10. The failure process of the asphalt mixture can be distinguished into three stages. At stage one, for the comparison group, the cumulative energy curve was at a low level and remained almost horizontal. After short-term aging as well as long-term aging at different times, the cumulative energy curve exhibited an increasing trend. This indicated that the aging effect exacerbated the formation of micro-cracks in the asphalt mixture at the early stage under the splitting load. At stage two, the cumulative energy curve for the comparison group displayed a gradual upward trend. After short-term aging, the cumulative energy curve first plateaued and then increased rapidly. After long-term aging at various times, the cumulative energy curve maintained a high rate of increase throughout this stage. The more rapid the increase in the curve, the more intense the cracking activities. The above results demonstrate that the aging effect accelerated the expansion of micro-cracks within the asphalt mixture into macro-cracks, and the long-term aging effect caused more intense cracking activities at this stage compared to short-term aging. At stage three, the cumulative energy curve of the comparison group exhibited an instantaneous steep increase. After short-term aging and long-term aging times of 24, 72, and 120 h, the cumulative energy curve also displayed a sudden increase, but the increment was smaller than that of the comparison group. After long-term aging for 168 h, the cumulative energy curve gradually increased without a precipitous increment. The above results demonstrate that the aging effect caused early damage to the asphalt mixture under a splitting load, resulting in earlier energy release and diminished energy surges at the final stage.

#### 3.2.4. Peak Frequency

**Figure 11:**
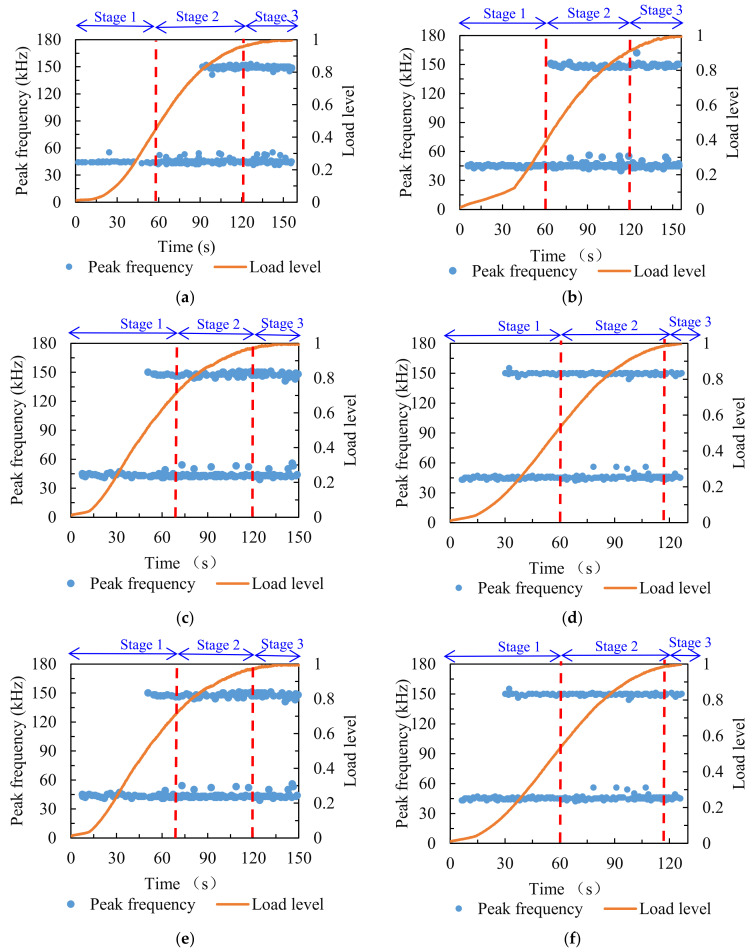
Peak frequency distribution of specimens treated with various aging conditions: (**a**) 0 h; (**b**) STA; (**c**) LTA of 24 h; (**d**) LTA of 72 h; (**e**) LTA of 20 h; (**f**) LTA of 168 h.

The variations of peak frequency versus time for the asphalt mixture subjected to various aging conditions are presented in Figure 11. The stage divisions were aligned with those in the energy and cumulative energy analysis for intuitive comparison. It can be seen that after various aging times, there were two distinct peak frequency bands during the failure process of asphalt mixtures, which were concentrated around 45 and 150 kHz, respectively. According to the previous analysis, the peak frequency near 45 kHz represented the AE signal released by the cracking of the matrix, and the peak frequency near 150 kHz represented the AE signal generated by the debonding of the matrix from the aggregates.

For the comparison group, the peak frequency around 45 kHz was observed throughout the whole damage process, and the peak frequency around 150 kHz started to emerge from the middle of the second stage until the final fracture of specimens. After short-term aging, the low value of peak frequency (around 45 kHz) was found during the entire failure process, and the high value of peak frequency (around 150 kHz) was observed from the beginning of the second stage until the final collapse of the asphalt mixture. After long-term aging of varying duration, it was also observed that peak frequency around 45 kHz appeared throughout the failure process. With the increase in long-term aging duration, the peak frequency around 150 kHz emerged gradually earlier, and it was found at the initial loading stage when the long-term aging duration reached 168 h. The above results demonstrate that the matrix cracking continued throughout the loading process in all specimens, and the aging effect weakened the adhesion between the matrix and aggregates, advancing the appearance of cracking caused by debonding. The longer the aging duration, the more significant the effect on the adhesion between the matrix and the aggregates.

Combining the analysis of energy, cumulative energy and peak frequency, it can be inferred that the aging effect altered the damage characteristics of the comparison group, aggravating the formation of micro-cracks and accelerating the development of macro-cracks, weakening the adhesion between the matrix and the aggregates, contributing to the early damage of the specimens.

## 4. Conclusions

In the present study, the fracture characteristics of the asphalt mixture treated with various freeze–thaw (F–T) cycles and various aging times were investigated based on the acoustic emission technique, and the main conclusions are drawn as follows:

- The failure loads and failure strains of the asphalt mixture decreased as the number of F–T cycles increased; with the increase in aging time, the failure loads of asphalt mixture increased, while the failure strains decreased.
- The damage process for all specimens can be subdivided into three stages, and the fracture characteristics of the asphalt mixture in the comparison group were characterized by a sudden and pronounced fracture at the final stage.
- The F–T cycling effect altered the damage characteristics of the asphalt mixture, leading to early damage under the splitting load, intensifying the formation of micro-cracks, promoting the expansion of macro-cracks and advancing the debonding of the matrix from the aggregates.
- The aging effect also influenced the fracture characteristics of the asphalt mixture under splitting load, weakening the adhesion between the matrix and the aggregates, exacerbating the generation of micro-cracks at an early stage, accelerating the rapid accumulation of macro-cracks and leading to earlier damage to the asphalt mixture.
- The acoustic emission technique can monitor the damage evolution of the asphalt mixture in real-time, which helps to further clarify the mechanism of freeze–thaw cycling and aging effects on the asphalt mixture.

In future studies, more acoustic emission parameters need to be adopted to evaluate the fatigue performance of asphalt mixtures, considering freeze–thaw cycling and aging effects.

## Figures and Tables

**Table 1:** Technical specifications of SBS modified asphalt.

Test Indicators	Test Values	Technical Requirements	Test Methods
Penetration (25 °C, 0.1 mm)	63.7	60~80	T0604
Softening point (°C)	62.2	≥55	T0606
Ductility (5 °C, cm)	35.6	≥30	T0605
Density (g/cm^3^)	1.072	-	T0603
Flashing point (°C)	272	≥230	T0611
Elastic recovery (25 °C, %)	75.3	≥65	T0662

**Table 2:** Technical specifications of aggregates and mineral powder.

Test Indicators		Test Values	Technical Requirements	Test Methods
Coarse aggregate	Crushing value (%)	15.2	≤26	T0316
	Los Angeles abrasion value (%)	19.3	≤28	T0317
	Apparent specific gravity	2.97	≥2.6	T0304
Fine aggregate	Mud content (%)	1.2	≤3.0	T0333
	Apparent specific gravity	2.85	≥2.5	T0304
Mineral powder	Hydrophilic coefficient	0.6	≤1.0	T0353
	Apparent specific gravity	2.73	≥2.5	T0304

## Data Availability

The data presented in this study are available on request from the corresponding author.
